# Exploring prognosis and therapeutic strategies for HBV-HCC patients based on disulfidptosis-related genes

**DOI:** 10.3389/fgene.2024.1522484

**Published:** 2025-01-15

**Authors:** Chuankuo Zhang, Xing Zhang, Shengjie Dai, Wenjun Yang

**Affiliations:** Department of Hepatobiliary Surgery, The First Affiliated Hospital of Wenzhou Medical University, Wenzhou, Zhejiang, China

**Keywords:** disulfidptosis, hepatitis B virus, hepatocellular carcinoma, immunotherapy, prognosis model

## Abstract

**Background:**

Hepatocellular carcinoma (HCC) accounts for over 80% of primary liver cancers and is the third leading cause of cancer-related deaths worldwide. Hepatitis B virus (HBV) infection is the primary etiological factor. Disulfidptosis is a newly discovered form of regulated cell death. This study aims to develop a novel HBV-HCC prognostic signature related to disulfidptosis and explore potential therapeutic approaches through risk stratification based on disulfidptosis.

**Methods:**

Transcriptomic data from HBV-HCC patients were analyzed to identify BHDRGs. A prognostic model was established and validated using machine learning, with internal datasets and external datasets for verification. We then performed immune cell infiltration analysis, tumor microenvironment (TME) analysis, and immunotherapy-related analysis based on the prognostic signature. Besides, RT-qPCR and immunohistochemistry were conducted.

**Results:**

A prognostic model was constructed using five genes (*DLAT*, *STC2*, *POF1B*, *S100A9*, and *CPS1*). A corresponding prognostic nomogram was developed based on riskScores, age, stage. Stratification by median risk score revealed a significant correlation between the prognostic signature and TME, tumor immune cell infiltration, immunotherapy efficacy, and drug sensitivity. The results of the experiments indicate that *DLAT* expression is higher in tumor tissues compared to adjacent tissues. *DLAT* expression is higher in HBV-HCC tumor tissues compared to normal tissues.

**Conclusion:**

This study stratifies HBV-HCC patients into distinct subgroups based on BHDRGs, establishing a prognostic model with significant implications for prognosis assessment, TME remodeling, and personalized therapy in HBV-HCC patients.

## 1 Introduction

Hepatocellular carcinoma (HCC) ranks sixth (4.3%) among newly diagnosed cancer cases globally and is the third (7.8%) leading cause of cancer-related deaths worldwide (Bray et al., 2024). In China, liver cancer is the third most common cancer and the second leading cause of cancer-related mortality (Zheng et al., 2024). In African and Asian populations, the primary etiological factor among hepatocellular carcinoma patients is chronic infection with hepatitis B virus (HBV) (Llovet et al., 2021). Over time, patients often progress from chronic hepatitis B to cirrhosis and eventually HBV-HCC. Due to the marked heterogeneity of hepatocellular carcinoma, the early stages of the disease often present with subtle symptoms, progress rapidly, and make prognosis challenging. Many patients are diagnosed at an advanced stage when symptoms appear, Once the opportunity for surgical intervention is missed, non-surgical treatment options generally offer limited efficacy for most patients (Yang et al., 2019). Given the high incidence, mortality, and significant heterogeneity of HBV-HCC, it is essential to identify more efficient diagnostic biomarkers and new therapeutic targets.

Liu et al. (2023) discovered a novel metabolism-related form of programmed cell death called disulfidptosis. They found that under glucose deprivation, cells with high expression of solute carrier family 7 member 11 (*SLC7A11*) experience accelerated depletion of cytoplasmic nicotinamide adenine dinucleotide phosphate (NADPH). The lack of NADPH prevents cells from reducing cystine to cysteine, leading to the accumulation of disulfides. This accumulation causes disulfide stress within the cell, resulting in aberrant disulfide bonding between actin cytoskeleton proteins, leading to actin network collapse and cell death. Inducing disulfidptosis by exploiting cancer metabolism weaknesses could provide new therapeutic approaches. Currently, disulfidptosis-related genes have been used to screen for prognostic markers and potential therapeutic targets in various malignancies such as colorectal adenocarcinoma (Li et al., 2024) and lung cancers. HBV-HCC, as one of the etiological types of hepatocellular carcinoma, is also the most prevalent type of this cancer in Asia and Africa (Zheng et al., 2024; Llovet et al., 2021). However, research related to HBV-HCC is still lacking. Furthermore, compared to tumors of other origins, hepatocellular carcinoma exhibits a high degree of heterogeneity (Yang et al., 2019). This indicates that we need to explore in greater depth and with more specificity the therapeutic value that disulfide death-related genes can offer to patients.

This study aims to identify subtypes of HBV-HCC patients with different prognoses, tumor immunity, somatic mutations, and clinical characteristics based on the expression of disulfidptosis-related genes (DRGs). We constructed a prognostic model for HBV-HCC that includes five genes: *DLAT*, *STC2*, *POF1B*, *S100A9*, and *CPS1*. We analyzed the tumor microenvironment of different risk groups and assessed the efficacy of immunotherapy and drug treatment based on risk scores. In summary, this study provides a new method for predicting the prognosis of HBV-HCC patients and offers new biomarkers for personalized treatment.

## 2 Materials and methods

### 2.1 Multiomics data collection and processing

The screening criteria for our study population included: 1. Positive results for serum hepatitis B virus surface antigen (HBsAg), positive HBV-DNA results, or a history of hepatitis B; 2. Pathological results indicating primary hepatocellular carcinoma. Patients who met at least one of the criteria in point 1 and also met the criteria in point 2 were identified as HBV-HCC patients in our study. We downloaded transcriptome data for 424 liver cancer cases from The Cancer Genome Atlas database (TCGA, https://gdc.cancer.gov/). After screening, we obtained 23 samples of adjacent normal tissue and 226 samples of HBV-HCC tumor tissue. We also obtained clinical data for 377 patients and gene mutation data (Simple Nucleotide Variation, SNV) for 368 liver cancer patients from the TCGA database. Additionally, we downloaded the GSE45114 dataset from the Gene Expression Omnibus database (GEO, https://www.ncbi.nlm.nih.gov/geo/), which includes RNA transcriptome data for 19 HBV-HCC samples. And we downloaded the GSE14520 (Roessler et al., 2010) dataset along with the corresponding follow-up data, which were then processed and filtered for analysis. The GSE14520 dataset contains the results of gene expression profiling conducted by Roessler et al. on tumor and paired non-tumor samples, as well as normal liver samples from 64 patients. From the UCSC Xena server (https://xena.ucsc.edu/), we acquired copy number variation (CNV) data. We have collected 118 disulfidptosis-related genes (Supplementary Data 2) from previous studies (Liao et al., 2023; Liu et al., 2023).

### 2.2 Identification and CNV analysis of disulfidoptosis-related genes in HBV-HCC patients

We used the “edgeR” R package to identify differentially expressed genes (DEGs) between HBV-HCC samples and normal tissues with a history of HBV in the TCGA database under the criteria |log2FC|>0.585 and Padj<0.05. By intersecting the genes included in GSE45114 with DRGs and DEGs, we define the resulting gene set as BHDRGs refers to the disulfidptosis-related genes in hepatitis B virus-associated hepatocellular carcinoma. We merged the TCGA HBV-HCC transcriptome data with the GSE45114 HBV-HCC transcriptome data using the “sva” and “limma” R packages and averaged gene expression levels for duplicate samples. Subsequently, we reassessed the differential expression of BHDRGs using the Wilcoxon rank-sum test. We evaluated the prognostic significance of BHDRGs using Kaplan-Meier (K-M) survival curves and univariate Cox analysis. Finally, we visualized the results.

To reveal the genomic alterations of BHDRGs, we processed CNV data of HBV-HCC patients and assessed the copy number variation status of BHDRGs in HBV-HCC patients.

### 2.3 Identification of different subtypes of HBV-HCC patients based on BHDRGs

Using the expression levels of BHDRGs and survival data, we performed an initial consensus clustering analysis of the samples using the “ConsensusClusterPlus” R package with the PAM algorithm. The optimal number of clusters (K) was determined using clustering heatmaps and the PAC method, and samples were divided into different subtypes. We evaluated the distinction between subtypes using principal component analysis (PCA). Kaplan-Meier survival analysis was performed on different subtypes using the “Survival” and “Survminer” R packages, and heatmaps were drawn combining clinical characteristics. To evaluate the differences in enriched metabolic pathways between subtypes, we performed GSVA (Gene Set Variation Analysis) using the “GSVA” and “GSEABase” R packages with files “c2.cp.kegg.symbols.gmt” and “c5.go.symbols.gmt” from MSigDB. We then assessed immune infiltration levels between subtypes using ssGSEA analysis. To explore potential reasons for the existence of different subtypes in HBV-HCC patients, we identified differential genes (interGenes) between groups using the “limma” R package under the conditions |log2FC| > 1 and P < 0.05.

### 2.4 GO and KEGG enrichment analysis

To analyze the relevant biological functions and structures of interGenes and to identify the corresponding enriched pathways, we utilized the R packages “ClusterProfiler,” “org.Hs.eg.db,” and “enrichplot” to conduct GO and KEGG analyses of the upregulated and downregulated genes of interGenes. The results were subsequently visualized using R.

### 2.5 Construction and validation of prognostic model and subtypes based on interGenes

We conducted a univariate Cox regression analysis on interGenes to identify genes significantly associated with prognosis (uniSigGenes). To investigate more thoroughly the interactions and consistency among BHDRGs and to reveal the finer differences among these subtypes, we performed a more refined clustering analysis of the patients using uniSigGenes.Using the “limma”, “ConsensusClusterPlus”, “survminer”, and “survival” R packages, we performed clustering analysis on the samples based on the expression levels of uniSigGenes using the PAM algorithm, determined the optimal K value and divided the samples into different gene subtypes (genecluster) according to the PAC method and clustering analysis results. Kaplan-Meier survival analysis was performed on gene subtypes combined with survival data.

Next, we randomly divided the samples into validation and test groups in a 1:1 ratio. Using the “glmnet” R package, we performed 10-fold cross-validation and 1000-cycle Least Absolute Shrinkage and selection Operator (LASSO) analysis in the test group, followed by multivariate COX regression analysis to determine the signature genes and establish the prognostic model. We calculated risk scores for samples based on the expression levels and related coefficients of the signature genes. The risk score (RS) formula is as follows:

$$\text{RS}=\left(\beta_{1}\times \text{expression of }{\text{gene}}_{1}\right)+\left(\beta_{2}\times \text{expression of }{\text{gene}}_{2}\right)+...+\left(\beta_{n}\times \text{expression of }{\text{gene}}_{n}\right).$$

We performed Kaplan-Meier survival analysis and time-dependent receiver operating characteristic (ROC) curve analysis for high and low-risk groups, and calculated the area under the ROC curve (AUC) to evaluate the model’s performance. The risk score formula was applied to the validation set and the total dataset. Patients were grouped based on risk scores, and heatmaps of signature gene expression, risk score distribution, and survival curves were drawn to validate the prognostic value of the risk scores. We selected the GSE14520 dataset for external validation of our model. The Kaplan-Meier survival curve and the ROC curve were used to evaluate the model’s performance within this external dataset. Additionally, we collected risk models from previous relevant literature to compare with our proposed BHDRGs model. The efficacy of each model was assessed using Kaplan-Meier survival analysis and ROC curve analysis.

Using the “rms” and “regplot” R packages, we constructed an HBV-HCC prognostic nomogram with the help of riskScores, age, gender, stage (American Joint Committee on Cancer-Tumor Node Metastasis staging), AFP (Alpha-Fetoprotein), grade (tumor pathological grading). We plotted calibration curves for 1, 3, and 5 years to verify the accuracy of the nomogram, ROC curves to validate the discrimination of the nomogram, and decision curve analysis (DCA) curves to assess the clinical applicability of the nomogram.

### 2.6 Immune cell infiltration analysis, TME analysis, immunotherapy-related analysis, and TMB analysis

We conducted an analysis of immune cell infiltration based on risk scores, tumor microenvironment (TME) analysis, immunotherapy-related analysis, and tumor mutation burden (TMB) analysis.

First, we used the CIBERSORT algorithm (https://cibersort.stanford.edu/) (Newman et al., 2015) combined with the LM22 signature matrix (Newman et al., 2015) to analyze the infiltration of 22 immune cell types in the TME. In the “Configure Basic CIBERSORT Options” section, since the data had already been normalized, we chose to reject the normalization of the uploaded data, and all other configurations were set to default. Based on the results of the CIBERSORT algorithm, we generated visualizations illustrating the infiltration of immune cells in high and low-risk groups. Furthermore, to assess the levels of stromal and immune cells in the TME in HBV-HCC, we employed the “estimate” R package to evaluate the differences in immune scores, stromal scores, and estimate scores between risk groups.

Subsequently, we performed differential analysis of immune checkpoint gene expression in high and low risk groups. We also assessed the effectiveness of immunotherapy between high and low risk groups using the tumor immune dysfunction and exclusion (TIDE) scores (http://tide.dfci.harvard.edu/). IMvigor 210 is a clinical trial cohort that investigates the efficacy of anti-PD-L1 immunotherapy in patients with metastatic urothelial carcinoma. We obtained transcriptomic data from the IMvigor-210 and selected patient samples with complete immunotherapy data. Using data from the IMvigor-210, we evaluated the ability of our model to predict patient responses to immunotherapy. Additionally, we evaluated the TMB of patients using TCGA somatic mutation data, comparing the TMB status between different risk groups. All results were visualized using the R package.

### 2.7 Drug sensitivity analysis

We obtained drug half-maximal inhibitory concentration (IC50) data from the Genomics of Drug Sensitivity in Cancer (GDSC) database. Subsequently, we performed drug sensitivity analysis using the “pRRophetic” R package to predict differences in drug therapy based on tumor gene expression levels between the high-risk and low-risk groups. We also conducted a t-test to compare the IC50 values between these two groups. The analysis results were visualized using the “ggplot2” R package.

### 2.8 RT-qPCR and immunohistochemistry

We collected tumor and adjacent non-tumor tissues from 20 patients with HBV-HCC at the First Affiliated Hospital of Wenzhou Medical University. The study protocol was approved by the Ethics Committee of the First Affiliated Hospital of Wenzhou Medical University (reference number: 2020-074). Written informed consent was obtained from all participants, and the study adhered to the standards outlined in the Declaration of Helsinki.

#### 2.8.1 RT-qPCR

Total RNA from cells was extracted using TRIzol reagent. According to the manufacturer’s instructions, RNA was reverse transcribed into cDNA using the HiScript IV All-in-One Ultra RT SuperMix. RT-qPCR was performed using TB Green Premix Ex Taq II. The reaction conditions were set as follows: 95°C for 30 s, followed by 40 cycles of 95°C for 5 s and 60°C for 30 s. GAPDH mRNA was used as an internal control, and relative mRNA levels were determined using the 2^−ΔΔCT^ method. The primer sequences were as follows: DLAT forward primer: 5′-CCG​CCG​CTA​TTA​CAG​TCT​TCC-3′; DLAT reverse primer: 5′-CTC​TGC​AAT​TAG​GTC​ACC​TTC​AT-3′. GAPDH forward: GCT​GAG​AAC​GGG​AAG​CTT​GT, GAPDH reverse: GCC​AGG​GTG​CTA​AGC​AGT​T.

#### 2.8.2 Immunohistochemistry

Tissues were fixed in formalin, embedded in paraffin, and sectioned into 5 μm slices. The primary antibody for IHC staining was DLAT (13426-1-AP, Proteintech, Chicago, United States). The antibody dilution ratio and subsequent experiments were performed according to the manufacturer’s instructions. IHC staining was carried out using DAB solution.

### 2.9 Statistical analyses

Unless otherwise specified in the text, we used the Wilcoxon rank-sum test to compare differences in continuous variables between the two groups, and the Kaplan-Meier curve along with the log-rank test to evaluate survival outcomes among different groups. Cox proportional hazards regression analysis was employed to identify independent prognostic factors. The Kruskal–Wallis rank-sum test was utilized to assess differences in gene expression across different patient classifications. Chi-square tests were conducted to compare the associations of categorical variables. Lastly, Pearson correlation analysis was used to evaluate the linear correlation between two continuous variables. R version 4.2.0 was used for statistical analyses. Perl was used to organize the data. To minimize batch effects within the dataset, we applied the ComBat function from the “sva” R package for data adjustment (Leek et al., 2012). For statistical significance in the entire text, numbers, and figure legends, the following terms were used: ***p < 0.001, **p < 0.01, *p < 0.05.

## 3 Result

### 3.1 Identification of disulfidptosis-related gene sets in HBV-HCC

**FIGURE 1:**
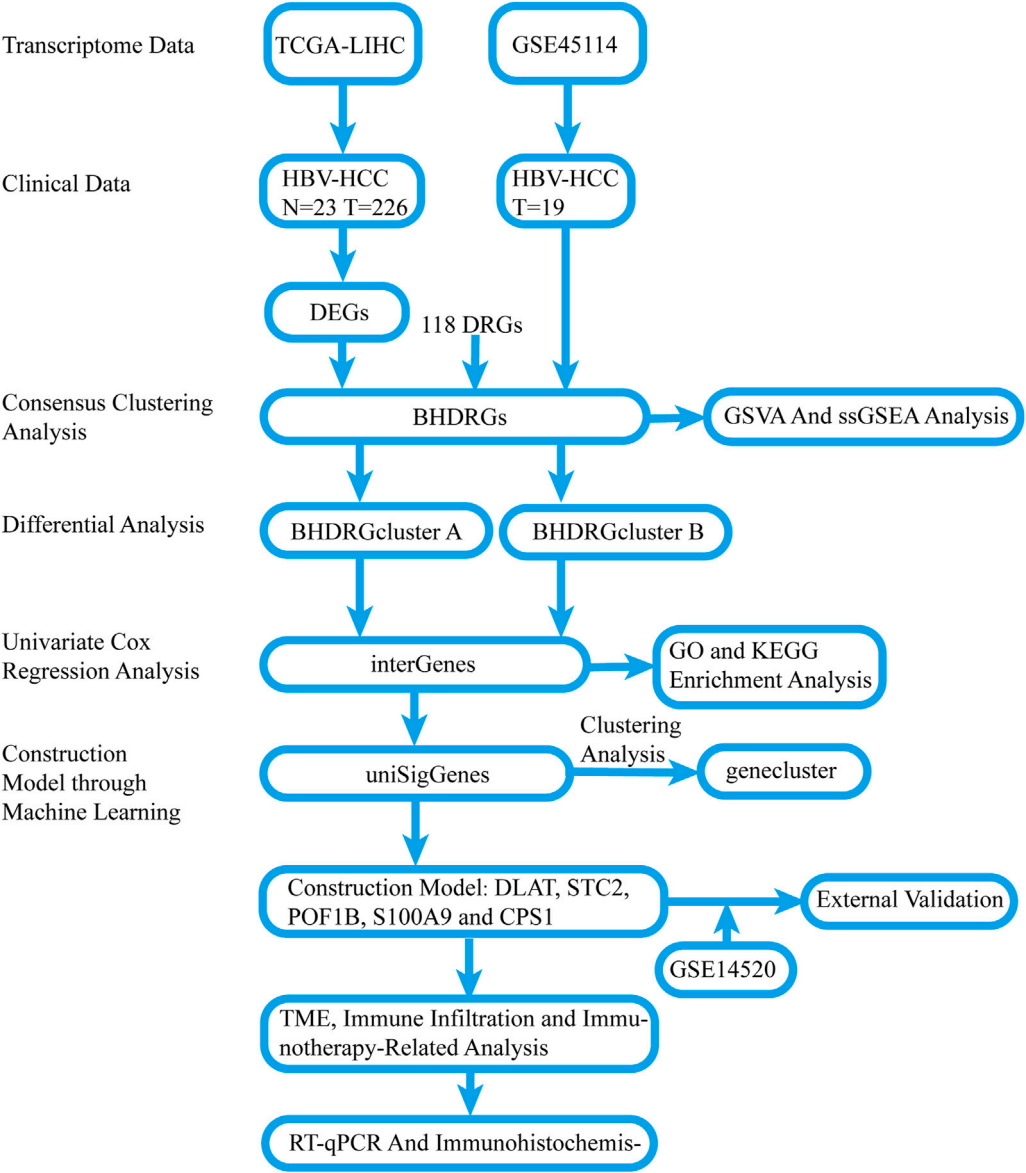
Flow chart of the study. N: cancer-adjacent normal tissue, T: tumor tissue.

**FIGURE 2:**
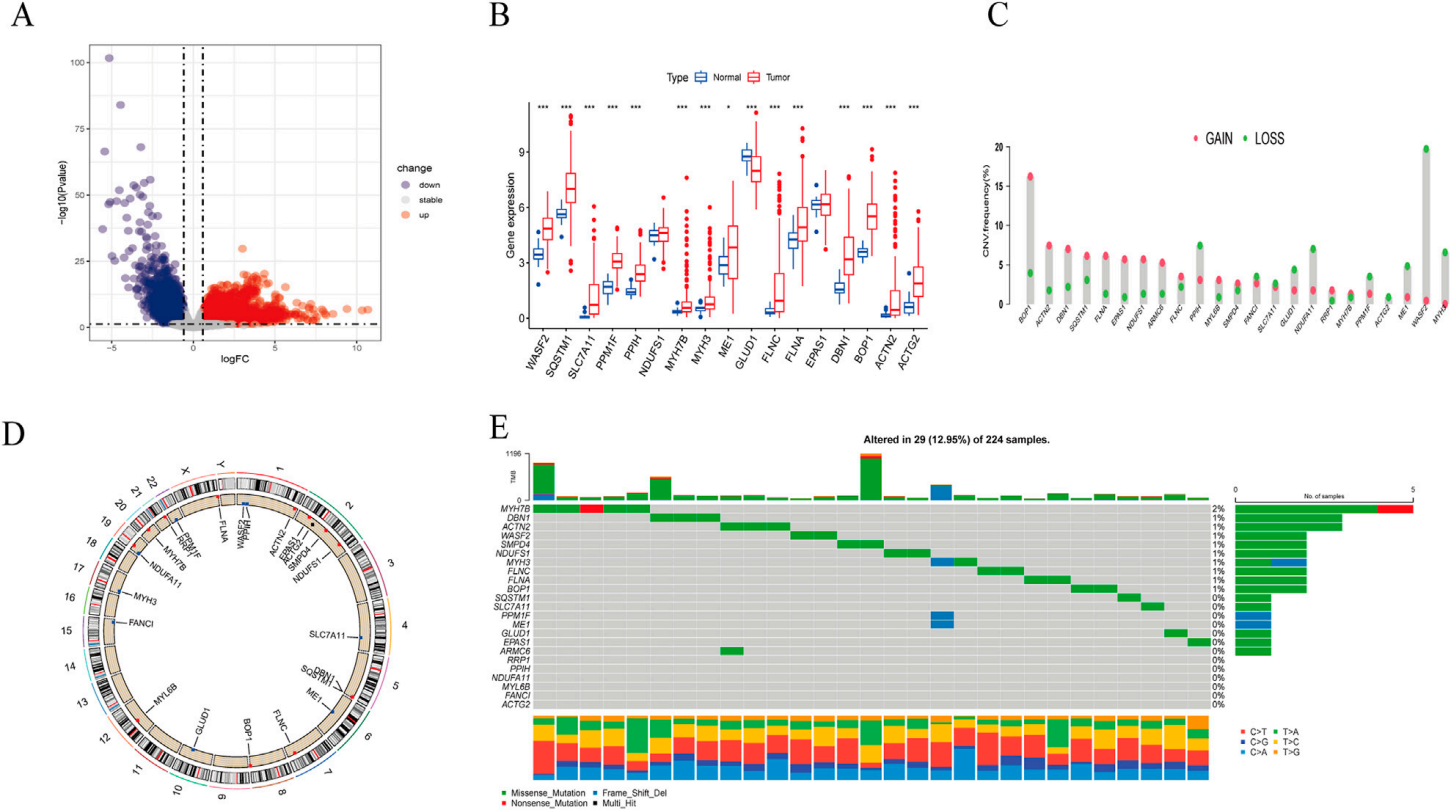
Expression and genetic alteration of BHDRGs in HBV-HCC. **(A)** DEGs between HBV-HCC samples and normal tissues with a history of HBV in the TCGA. **(B)** The expression of 17 BHDRGs in HBV-HCC and normal tissues. **(C)** The copy number variation frequency of BHDRGs in HBV-HCC. **(D)** Variation in the copy number of the BHDRGs at various places on the chromosome. **(E)** The mutation frequency of BHDRGs in HBV-HCC. *p < 0.05, p < 0.01. ***p < 0.001.

The overall process of this study is illustrated in Figure 1. Comparing the gene expression between HBV-HCC samples from the TCGA and normal tissue samples with a history of HBV infection, we identified 8,110 DEGs, including 2,620 downregulated genes and 5,490 upregulated genes (Figure 2A). We intersected these genes to obtain 17 BHDRGs and plotted a box plot of their differential expression in HBV-HCC tumor and non-tumor tissues (Figure 2B). We also analyzed the copy number variation frequency of the BHDRGs in the samples (Figure 2C), finding that the increase in *BOP1* copies was significantly higher than the deletion frequency, while the deletion frequency of *WASF2* copies was significantly higher than the increase. Figure 2D illustrates the locations of BHDRGs on the chromosomes along with their copy number variations.

**FIGURE 3:**
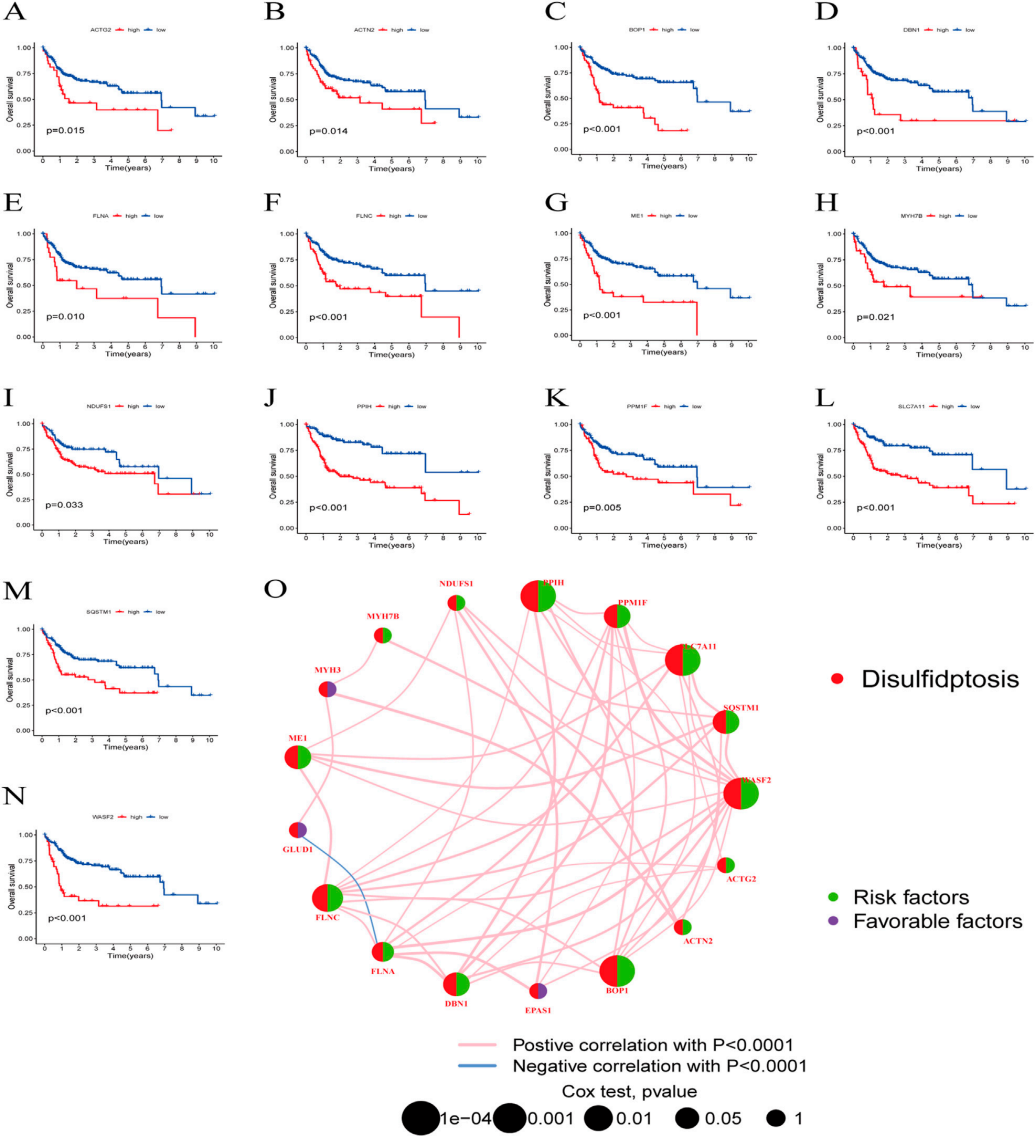
The K-M analysis revealed that 14 genes within the BHDRGs were significantly associated with HBV-HCC patient prognosis. **(A)** ACTG2. **(B)** ACTN2. **(C)** BOP1. **(D)** DBN1. **(E)** FLNA. **(F)** FLNC. **(G)** ME1. **(H)** MYH7B. **(I)** NDUFS1. **(J)** PPIH. **(K)** PPM1F. **(L)** SLC7A11. **(M)** SQSTM1. **(N)** WASF2. **(O)** Prognostic network of BHDRGs.

Next, we examined the somatic mutation data in the samples, finding mutations in 29 out of 224 HBV-HCC, with *MYH7B* having the highest mutation rate (Figure 2E). We then combined the GSE45114 data with the TCGA data, obtaining 240 HBV-HCC samples. The univariate Cox analysis results of BHDRGs in HBV-HCC samples are shown in Table 1. We evaluated the association between significant genes from univariate analysis and patient prognosis using K-M survival analysis (Figures 3A–N). The results indicating that *MYH3*, *GLUD1*, and *EPAS1* are protective factors, while *WASF2*, *SQSTM1*, *SLC7A11*, *PPM1F*, *PPIH*, *NDUFS1*, *MYH7B*, *ME1*, *FLNC*, *FLNA*, *DBN1*, *BOP1*, *ACTN2*, and *ACTG2* are risk factors for the prognosis of HBV-HCC patients. As shown in Figure 3O, the prognostic network outlines the interactions, interconnections, and prognostic value of BHDRGs in HBV-HCC patients.

**TABLE 1:** UniCOX and K-M analysis of BHDRGs.

BHDRGs	HR	HR.95L	HR.95H	P value	K-M
WASF2	1.78043622910376	1.33708070924039	2.37080166066124	7.87677978821637e-05	3.55311100652678e-07
SQSTM1	1.33434998953886	1.12369068176839	1.58450178814362	0.00100117663758269	0.00066210872624306
SLC7A11	1.35762116142048	1.17961462647855	1.56248928808126	2.01192813861604e-05	3.65573307781197e-06
PPM1F	1.62514167477145	1.12844017346628	2.34047451090492	0.00907394105517528	0.00538794975274259
PPIH	2.18143064042544	1.6213976377083	2.93489982242286	2.56912300734912e-07	2.76170289970068e-07
NDUFS1	1.21898491734471	0.865744634008937	1.71635395744025	0.256705454486927	0.0329665526095082
MYH7B	1.14261723745651	0.937396363715234	1.39276639196496	0.186861745676822	0.020684402272597
MYH3	0.98503791298912	0.763288530555492	1.27120957696012	0.907771739843421	0.136449672094353
ME1	1.2325939115488	1.08366386526288	1.40199170562785	0.00145817633742628	9.11067761032447e-05
GLUD1	0.892011843294768	0.721576548346996	1.10270369845154	0.290836039536036	0.123585664108539
FLNC	1.22894947368026	1.10570641068638	1.36592932288548	0.000131489245536833	0.000219279329147892
FLNA	1.18987568102055	1.03015079711054	1.37436590861773	0.0180849428266241	0.0100213101547357
EPAS1	0.957076779594197	0.733693140720981	1.24847284402615	0.746308276377221	0.256481526551826
DBN1	1.24577417569125	1.07092137164908	1.44917576388397	0.00440032456372329	0.000461759687809371
BOP1	1.50442492833529	1.23080057477749	1.83887984079452	6.67787043017316e-05	1.70019810341593e-07
ACTN2	1.07559076254638	0.947829902277709	1.22057289572211	0.258696643330815	0.0137511891716948
ACTG2	1.17245421855475	0.961409896557714	1.42982602896921	0.11611473165463	0.0149672918688798

### 3.2 Identification and validation of HBV-HCC subtypes

**FIGURE 4:**
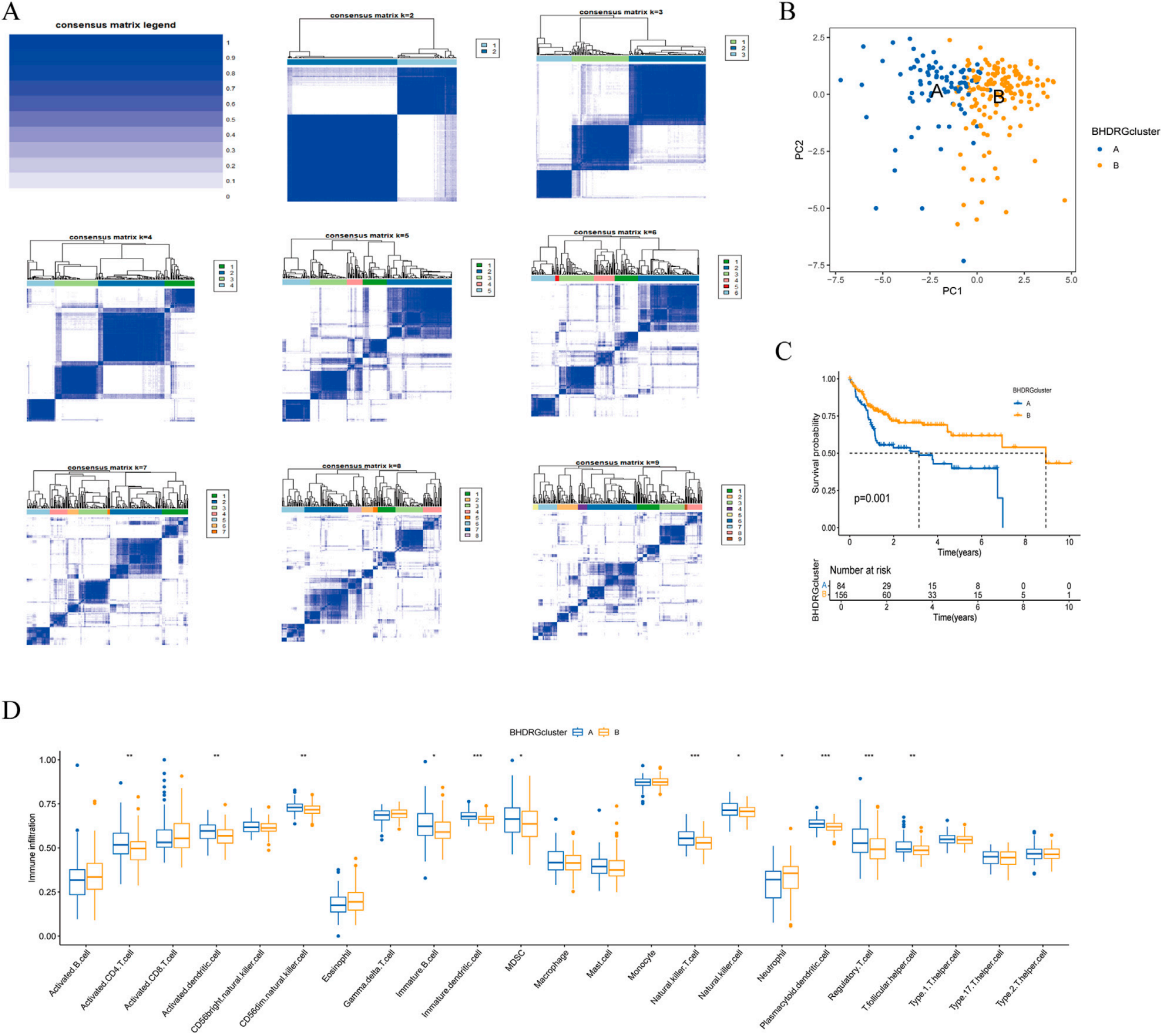
**(A)** The clustering analysis of HBV-HCC samples based on gene expression profiles. **(B)** The principal component analysis of subtypes. **(C)** Kaplan-Meier survival analysis shows the two clusters. **(D)** The differential analyse between immune cells mong the two clusters. *p < 0.05, **p < 0.01, *** k p < 0.001.

To clarify the relationship between BHDRGs and HBV-HCC, we performed consensus clustering analysis on HBV-HCC samples. Using the PAC algorithm in combination with clustering results (Figure 4A), we divided the samples into BHDRG cluster A and BHDRG cluster B. Principal component analysis (PCA) was then used for dimensionality reduction and visualization (Figure 4B). The PCA results showed good discrimination between the two subtypes. Subsequently, we conducted KM survival curve analysis (Figure 4C), showing that the 1 year, 3 year, and 5 year survival rates of group B were significantly better than those of group A. Combined with age, sex and T stage, we plotted a heatmap of BHDRG subtypes in HBV-HCC patients (Supplementary Figure 3A).

GSVA shows the differences between the top20 most significant pathways in cluster A and cluster B (Supplementary Figure 3B). The results indicate that, compared to the BHDRG cluster A, pathways such as linoleic acid metabolism, alpha-linolenic acid metabolism, maturity-onset diabetes of the young, alanine, aspartate, and glutamate metabolism, nitrogen metabolism, and glycine, serine, and threonine metabolism, as well as signaling pathways including the peroxisome proliferator-activated receptor signaling pathway, olfactory transduction, and neuroactive ligand-receptor interaction, are more active in the BHDRG cluster B. In contrast, gene sets related to lysosomes, pathogenic *Escherichia coli* infection, endocytosis, epithelial cell signaling in *Helicobacter pylori* infection, neurotrophin signaling pathway, pathways in cancer, small cell lung cancer, pancreatic cancer, and colorectal cancer show higher expression levels in the BHDRG cluster A.

And the boxplot showed the difference of immune cell infiltration in different subtypes (Figure 4F).In the BHDRG cluster A group, the infiltration levels of activated CD4 T cells, activated dendritic cells, CD56dim natural killer cells, immature B cells, immature dendritic cells, myeloid-derived suppressor cells (MDSCs), natural killer T cells, natural killer cells, plasmacytoid dendritic cells, regulatory T cells, and T follicular helper cells are significantly higher than those in the B group. Conversely, neutrophils exhibit higher infiltration levels in the BHDRG cluster B group.

### 3.3 Obtaining differential Genes, GO and KEGG enrichment analysis

**FIGURE 5:**
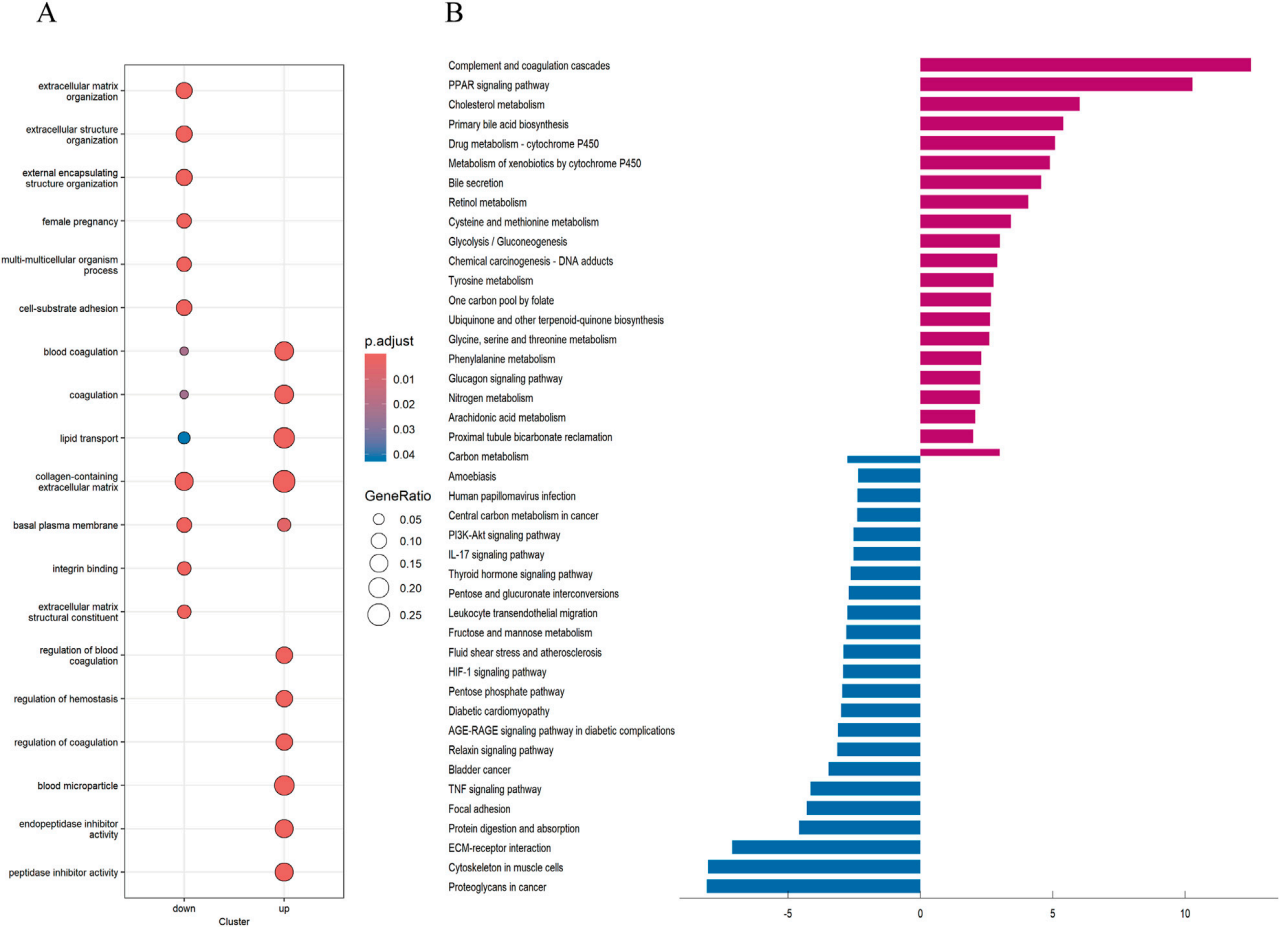
GO and KEGG analysis **(A)** Analysis of GO enrichment demonstrated the possible function of the interGene. **(B)** KEGG pathway enrichment analysis revealed the possible pathways.

To explore the potential reasons for the prognostic differences between subtypes, we performed differential analysis to obtain interGenes (Supplementary Data 1). And we performed GO and KEGG enrichment analyses to identify the functions and pathways enriched for the upregulated and downregulated genes of interGenes. The GO analysis demonstrated (Figure 5A) that the downregulated genes in interGenes were significantly enriched in collagen-containing extracellular matrix, extracellular matrix organization, extracellular structure organization, and external encapsulating structure organization. And the upregulated genes were primarily enriched in collagen-containing extracellular matrix, blood coagulation, lipid transport, and peptidase inhibitor activity. The KEGG enrichment analysis results indicated (Figure 5B) that the downregulated genes were involved in biological pathways such as Proteoglycans in cancer, Cytoskeleton in muscle cells, ECM-receptor interaction, protein digestion and absorption, and focal adhesion. Meanwhile, the upregulated genes showed significant enrichment in the complement and coagulation cascades, PPAR signaling pathway, and various metabolic pathways.

### 3.4 Consensus clustering and risk model construction based on differential genes in disulfidptosis

**FIGURE 6:**
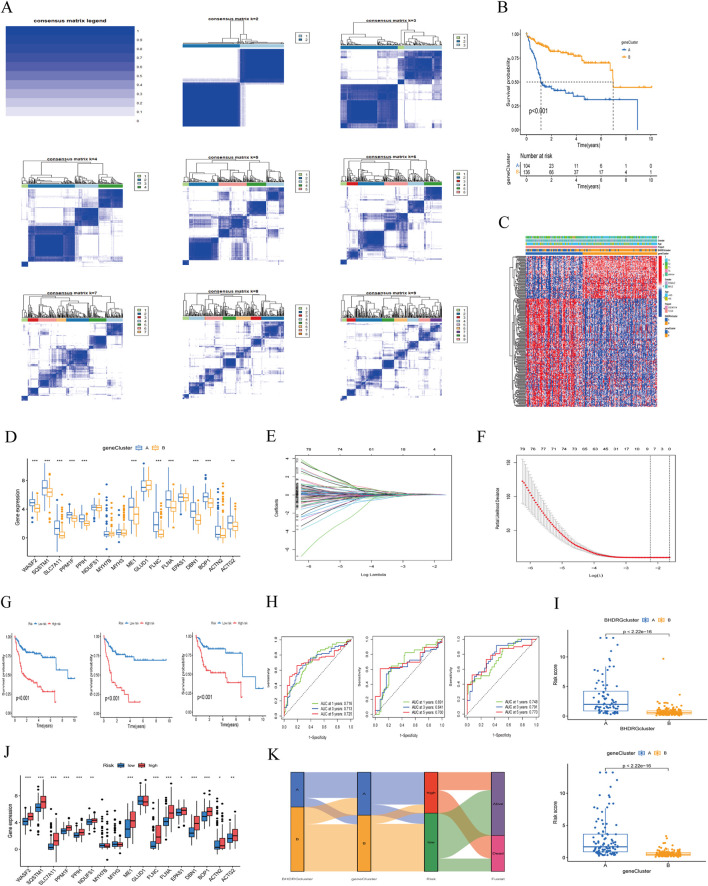
**(A)** Concordance matrix of subtypes. **(B)** Kaplan-Meier survival curve of the geneClusters. **(C)** A heatmap illustrated the expression patterns. **(D)** Expression of BHDRGs between geneCluster A and geneCluster **(B, E)** LASSO regression analysis. **(F)** Partial likelihood deviance on the prognostic genes. **(G)** Kaplan-Meier survival curves for patients in the high- and low-risk groups defined by the prognostic model in the all set, test set, and train set. **(H)** ROC curve analyses for 1 year, 3 year, and 5 year survival rates in the all set, test set, and train set. **(I)** The differences in risk score in the BHDRGcluster and the geneCluster. **(J)** The expression of BHDRGs in the high- and low-risk groups. **(K)** Sankey diagram shows the construction of the prognostic model.

For more accurate sample typing, we conducted univariate Cox regression analysis on interGenes to identify uniSigGenes. Based on uniSigGenes, we performed another clustering analysis (Figure 6A). Both the clustering heatmap and PAC algorithm suggested that the optimal clustering model was achieved when K = 2, resulting in geneclusterA and geneclusterB. KM survival curve analysis (Figure 6B) showed that the prognosis of group B was significantly better than group A. We plotted a heatmap of gene clusters, considering patients’ age, sex, and T stage (Figure 6C). We also evaluated the differential expression of BHDRGs between gene clusters (Figure 6D).

In the test group, we conducted LASSO-Cox analysis on the expression levels of uniSigGenes (Figures 6E, F), identifying 9 genes (*DLAT*, *SLC2A1*, *STC2*, *SLC38A1*, *POF1B*, *S100A9*, *AP1M2*,*MMP9*, *CPS1*) with optimal λ values. We then performed multivariate regression analysis on these 9 genes to construct a new prognostic risk model, including key genes such as *DLAT*, *STC2*, *POF1B*, *S100A9*, and *CPS1*. The risk scoring formula for the model is as follows:

$$\begin{array}{l} \text{RS}=\text{expDLAT}*0.43798077\;5786097+\text{expSTC}2*0.24041938\;9109836+\text{expPOF}1B*0.17373046\;5002983+\\ \text{expS}100A9*0.19007896\;8663933-\text{expCPS}1*0.11323419\;4208144 \end{array}$$

We assigned risk scores to all samples based on the risk formula, dividing patients into high-risk and low-risk groups using the median risk score and conducting Kaplan-Meier survival analysis (Figure 6G). The survival curves of the total group, training group, and test group all showed that the survival rate of the low-risk group was significantly higher than that of the high-risk group. We plotted 1 year, 3 year, and 5 year ROC curves to evaluate the sensitivity and specificity of the gene signature and calculated the area under the ROC curve (AUC), indicating that our model has good predictive power (Figure 6H). And we validated patient groups and their prognostic differences in the GSE14520 dataset (Supplementary Figure 1). Additionally, we compared our model with previous risk models (Supplementary Figure 2). Compared to the Tang signature (Tang et al., 2020), Tian signature (Tian et al., 2024), Li signature (Li et al., 2022), and Weng signatures (Weng et al., 2023),BHDRGs signature demonstrated superior performance in predicting the prognosis of HBV-HCC patients.

We also evaluated the risk score differences between BHDRG subtypes and gene clusters (Figure 6I) and plotted heatmaps related to risk grouping (Supplementary Figure 4). We found that the risk score of genecluster-A was significantly higher than that of genecluster-B, and the risk score of BHDRG cluster A was higher than that of BHDRGcluster-B, consistent with our previous survival analysis results. Finally, we evaluated the expression differences of BHDRGs between high-risk and low-risk groups (Figure 6J). The genes *MYH7B*, *MYH3*, and *GLUD1* show no differential expression between the high-risk and low-risk groups. In contrast, the expression levels of genes such as *EPAS1*, *WASF2*, *SQSTM1*, *SLC7A11*, *PPM1F*, *PPIH*, *NDUFS1*, *ME1*, *FLNC*, *FLNA*, *DBN1*, *BOP1*, *ACTN2*, and *ACTG2* are significantly higher in the high-risk group compared to the low-risk group.

Next, we used Sankey diagrams (Figure 6K) to show the distribution of HBV-HCC patients in the clustering process. Most genecluster-A samples came from the BHDRGcluster-A group, which had worse survival data. In the risk scoring, they were mainly distributed in the high-risk score group, with deceased patients primarily concentrated in the high-risk group. The Sankey results were consistent with previous analysis results.

### 3.5 Construction and validation of a nomogram

**FIGURE 7:**
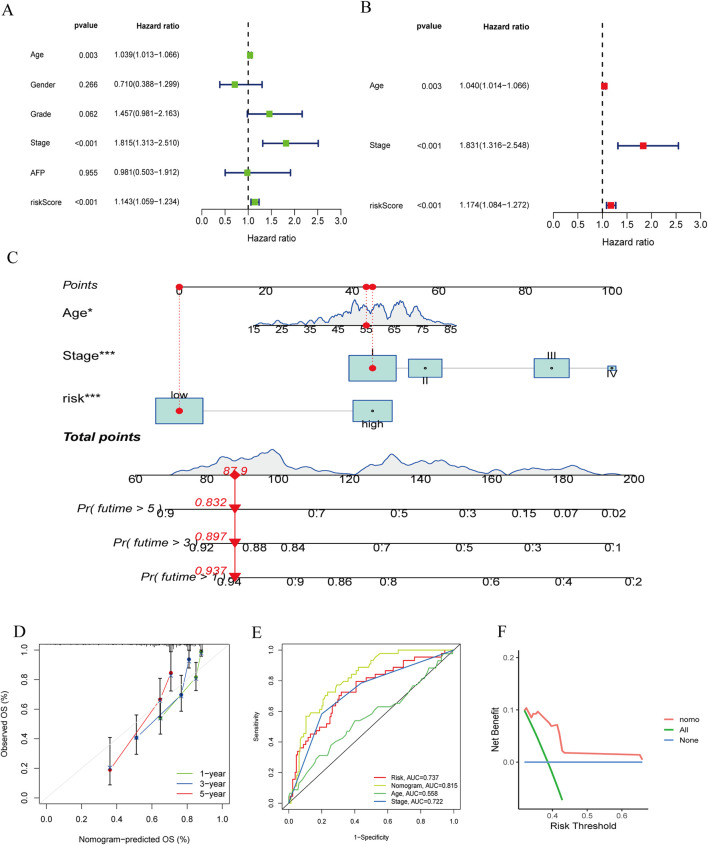
**(A, B)** The forest plots for univariate and multivariate. **(C)** The nomogram of the risk and clinical parameters (age, stage) of all sets. **(D)** The calibration curves displayed the accuracy of the nomogram in the l year, 3 year, and 5 year. **(E)** The ROC curve of the nomogram. **(F)** The DCA curve of the nomogram.

We conducted univariate and multivariate Cox regression analyses on the clinical features of all cases combined with riskScores (Figures 7A, B). Univariate Cox regression analysis showed that riskScore, age and stage were significantly associated with patient survival probability. Multivariate Cox regression analysis also found that riskScores, age and stage were independent predictors of prognosis. We then constructed a nomogram comprising riskScores, age, and stage (Figure 7C). We verified the accuracy of the nomogram by plotting calibration curves (Figure 7D). The ROC curve of the nomogram (Figure 7E) suggested that it had good discriminative power compared to other influencing factors. The DCA curve (Figure 7F) also showed that the nomogram had good applicability in clinical practice. Next, we compared our nomogram with those of Tian (Tian et al., 2024), Tang (Tang et al., 2020), and Li (Li et al., 2022). Our findings indicated that (Supplementary Figure 5), in the ROC analysis, calibration curves, and DCA curve, our nomogram consistently demonstrated superior performance. Parameters such as specificity, sensitivity, precision, positive and negative likelihood ratios, and Youden index can be found in Supplementary Data 3.

### 3.6 Tumor microenvironment, immune infiltration, and immunotherapy-related analysis

**FIGURE 8:**
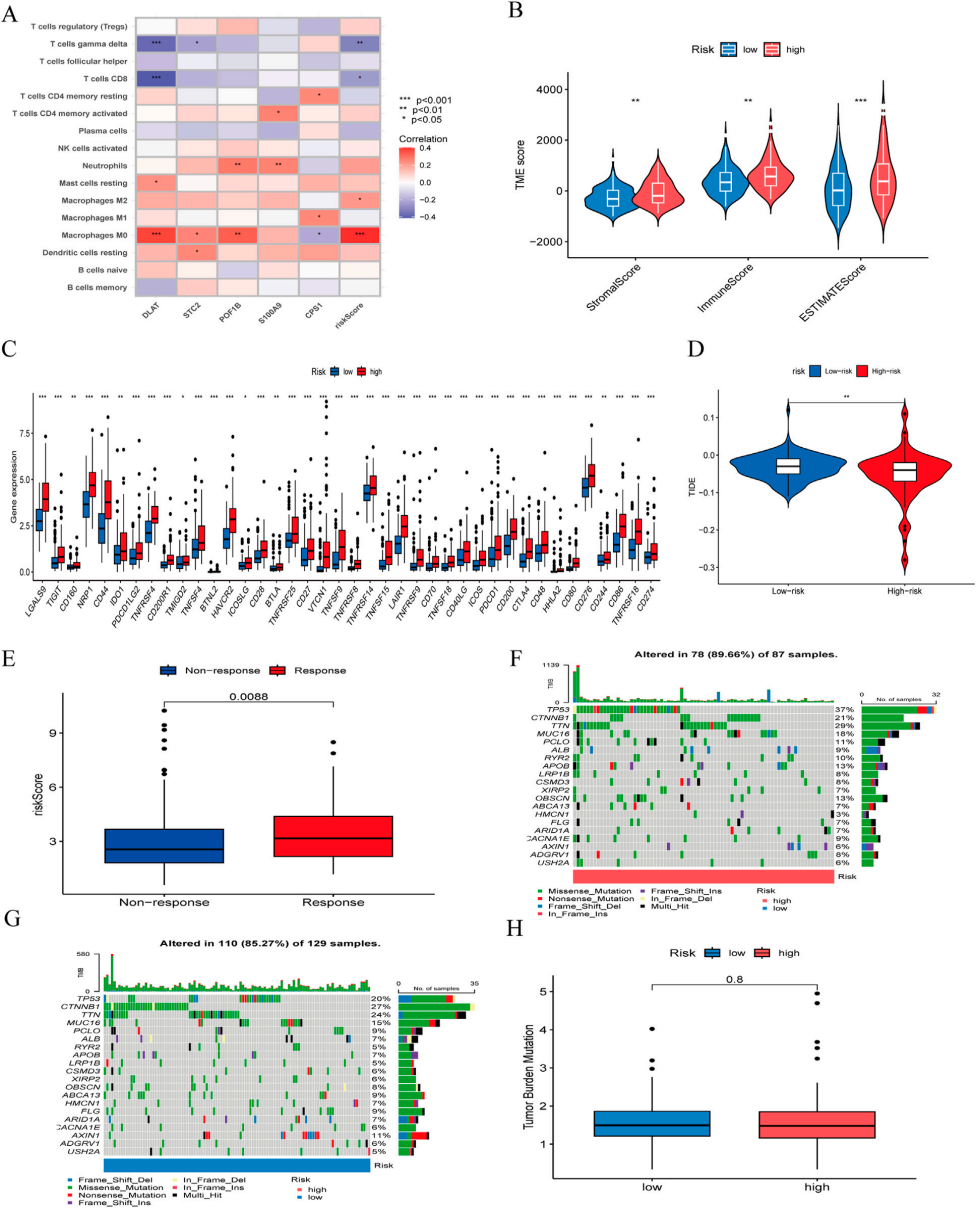
**(A)** correlation between the signature and immunecells. **(B)** comparison of ESTIMATE scores, Stromal scores, and Immune scores between the high-risk and low-risk groups. **(C)** Differential expression of immune checkpoints between the high-risk and low-risk groups. **(D)** Differences in TIDE score between high-risk and low-risk groups. **(E)** The ability of model to predict patient responses to immunotherapy in IMvigor 210. **(F)** The frequency of mutations in the high-risk and **(G)** low-risk groups. **(H)** Differences in TMB between high-risk and low-risk groups.

To assess the therapeutic value of the risk model for HBV-HCC patients, we explored immune cell infiltration between high-risk and low-risk groups. Immune infiltration analysis (Figure 8A) indicated that Macrophages M0 and M2 were positively correlated with risk scores, while T cells gamma delta and T cells CD8 were negatively correlated. In the tumor microenvironment scores (Figure 8B), StromalScore, ImmuneScore, and ESTIMATEScore were all significantly higher in the high-risk group than in the low-risk group. To assess the value of risk scores in immunotherapy, we examined the differential expression of immune checkpoint genes between high-risk and low-risk groups (Figure 8C). Results showed differential expression of 40 immune checkpoint genes between the two groups, with higher expression in the high-risk group, suggesting that high-risk patients might benefit more from immunotherapy. We used TIDE scores to evaluate the potential clinical efficacy of immunotherapy in different risk groups. Typically, higher TIDE scores indicate a higher likelihood of tumor immune escape and poorer efficacy of immune checkpoint inhibitors. The results suggested (Figure 8D) that high-risk patients had lower TIDE scores than low-risk patients, implying that high-risk patients might achieve better efficacy from immunotherapy. And the IMvigor 210 cohort revealed that (Figure 8E), patients who responded to anti-PD-L1 immunotherapy had higher risk scores in our risk model compared to those who did not benefit from the treatment. Additionally, we evaluated the mutation frequency of HBV-HCC patients between different risk groups (Figures 8F, G), showing that TP53 had the highest mutation frequency, with a significant difference in mutation frequency between high-risk and low-risk groups. However, we found that there was no significant difference in TMB between high-risk group and low-risk group (Figure 8H).

### 3.7 Drug sensitivity analysis

**FIGURE 9:**
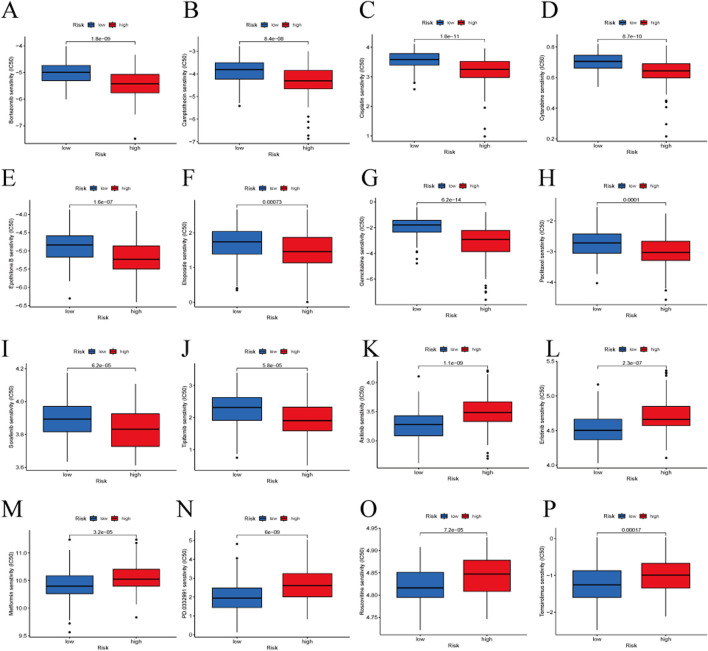
Drug sensitivity analysis between the high-risk and low-risk groups. **(A)** Bortezomib, **(B)** Camptothecin, **(C)** Cisplatin, **(D)** Cytarabine, **(E)** Epothilone B, **(F)** Etoposide, **(G)** Gemcitabine, **(H)** Paclitaxel, **(I)** Sorafenib, **(J)** Tipifarnib, **(K)** Axitinib, **(L)** Erlotinib, **(M)** Metformin, **(N)** PD 0332991 (Palbociclib), **(O)** Roscovitine, **(P)** Temsirolimus.

We assessed the predictive capability of our gene signature for drug treatment in HBV-HCC patients. High-risk groups showed greater sensitivity to drugs such as Bortezomib, Camptothecin, Cisplatin, Cytarabine, Epothilone B, Etoposide, Gemcitabine, Paclitaxel, Sorafenib, and Tipifarnib. Low-risk groups were more sensitive to Axitinib, Erlotinib, Metformin, PD 0332991 (Palbociclib), Roscovitine, and Temsirolimus (Figure 9).

### 3.8 RT-qPCR and immunohistochemistry in tumor tissues of HBV-HCC patients

**FIGURE 10:**
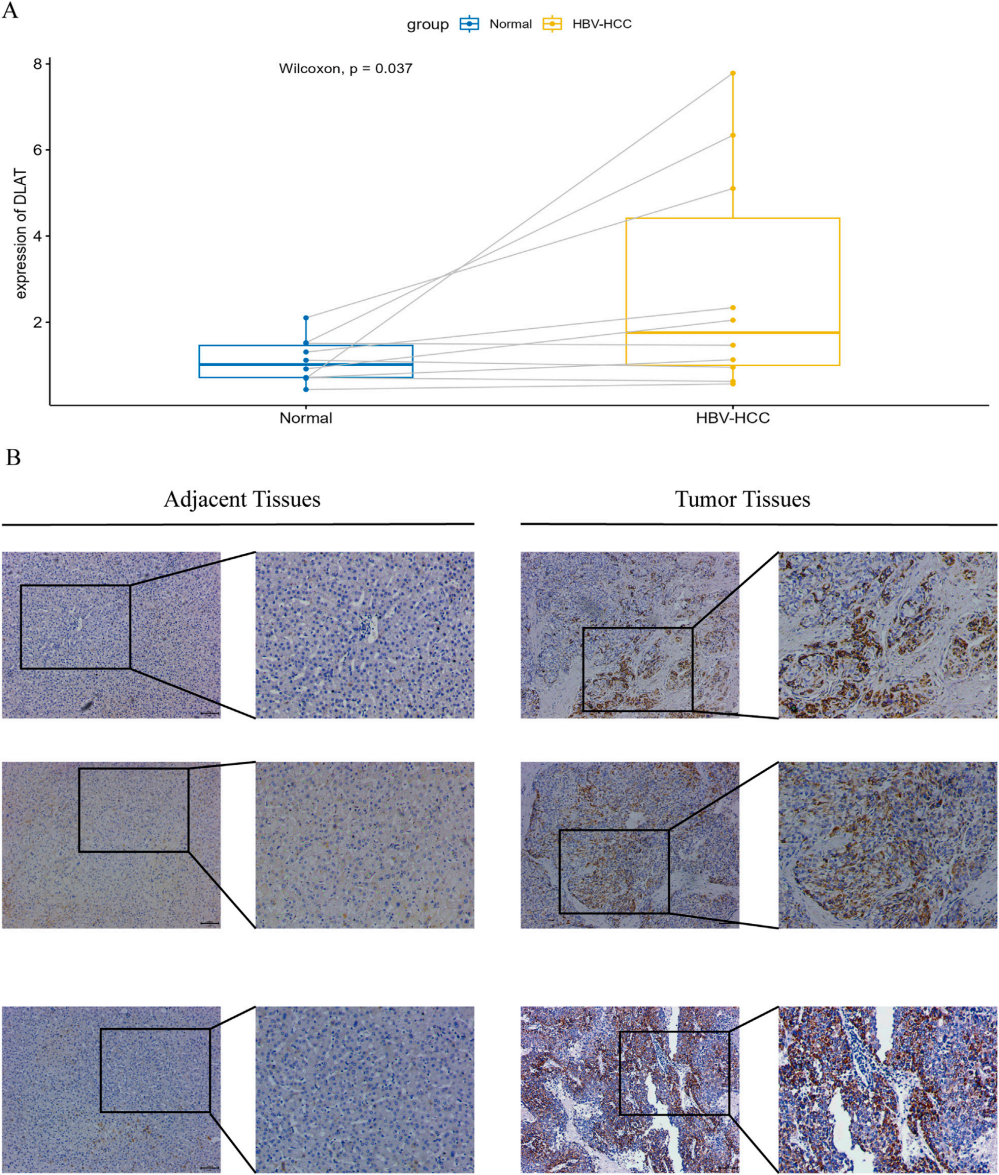
**(A)** Verify the mRNA expression levels of DLAT. **(B)** IHC staining was performed on tumor tissues and adjacent normal tissues to verify the expression of DLAT.

We conducted RT-qPCR analysis on 10 pairs of clinical samples from HBV-HCC patients. The results showed (Figure 10A) that the mRNA expression of *DLAT* in tumor samples was significantly higher than that in normal samples. Additionally, we conducted immunohistochemical analysis on another 10 pairs of clinical samples from HBV-HCC. Our immunohistochemical analysis revealed that DLAT protein expression in HBV-HCC tumor tissues was markedly elevated compared to adjacent normal liver tissues (Figure 10B).

## 4 Discussion

In recent years, the incidence of liver cancer has been on the rise (Siegel et al., 2022), with hepatocellular carcinoma (HCC) constituting the majority of liver cancer diagnoses and deaths (Llovet et al., 2021). A history of HBV infection has become one of the most common risk factors for HCC. Recently, Xiaoguang Liu and colleagues proposed a new regulatory form of cell death, disulfidptosis, which could be utilized in cancer treatment (Liu et al., 2023). Research has indicated the significant role of disulfidptosis in various tumors, including lung cancer (Wang et al., 2024), renal cell carcinoma (Xu et al., 2023). However, in the study of hepatocellular carcinoma (HCC), research related to disulfidptosis remains limited. Zhao et al. developed (Zhao et al., 2023) a tumor prognosis signature based on disulfidptosis to assist in clinical decision-making. However, they simultaneously noted that the impact of viral infection was not considered in their study. Given that the etiology of potential liver disease may influence prognosis, they suggested that the prognostic assessment could be biased. Therefore, the prognostic role of disulfidptosis and its value in guiding treatment strategies for HBV-HCC patients require further investigation. We conducted additional research on HBV-HCC based on the differential expression of BHDRGs and identified two subgroups within the HBV-HCC patient population that exhibited different clinical characteristics and survival outcomes. Additionally, we constructed a prognostic model for HBV-HCC patients to guide clinical treatment and facilitate risk stratification.

This study performed two clustering analyses based on BHDRGs, leading to the identification of two subtypes with distinct clinical characteristics and significantly different prognoses. The first clustering analysis provided a general classification of this population, while the second clustering analysis allowed for a more detailed subdivision of these subtypes, revealing finer differences among them. Notably, in the context of high-heterogeneity tumor research, the second clustering analysis aids in uncovering the pronounced heterogeneity of tumors and offers deeper insights into the roles and mechanisms of BHDRGs in the prognostic differences among the various subtypes. By employing the second clustering analysis, we can more accurately identify the different subtypes within tumors and design more targeted treatment strategies.

In the first clustering analysis, patients were categorized into two distinct subtypes, each exhibiting different clinical characteristics and prognostic outcomes. K-M survival analysis revealed a poorer prognosis for group A compared to group B in the BHDRGs subtypes. Immune infiltration analysis suggested higher immune cell abundance in group A samples, indicating a more active tumor immune microenvironment. To further explore the possible reasons for the survival performance differences between subtypes, GSVA pathway analysis was conducted. This analysis revealed the enrichment of pathways related to small cell carcinoma, prostate cancer, colorectal cancer, and bacterial infection in group A samples. In contrast, group B was mainly enriched in various metabolic pathways, such as those involving amino acids, arachidonic acid, and linoleic acid. Additionally, multiple ligand-receptor signaling pathways were enriched in group B. Mossmann et al. has indicated (Mossmann et al., 2023) that while the mechanisms and targets of metabolic reprogramming remain unclear, it is certain that metabolic reprogramming plays a significant role in the occurrence and progression of tumors. Furthermore, several studies (Kudo et al., 2020; Lin et al., 2024; Liu et al., 2018) suggest that metabolic reprogramming could serve as a new direction for cancer treatment.

To further analyze the connection between BHDRGs and HBV-HCC, GO functional enrichment analysis and KEGG pathway enrichment analysis were performed on interGene. The research findings indicate that interGenes are significantly enriched in various functions and structures, including extracellular structures, the extracellular matrix, and the cytoskeleton, and are involved in multiple metabolic and signaling pathways. Notably, Wu et al. indicated (Wu et al., 2023) the association of the extracellular matrix with the progression of various tumors, including hepatocellular carcinoma, pancreatic ductal adenocarcinoma, and breast cancer. Extracellular matrix stiffening promotes cell proliferation, epithelial-mesenchymal transition, cancer cell metastasis, and drug resistance (Pickup et al., 2014; Walker et al., 2018). The functional and pathway differences between these two subtypes are closely related to the mechanism of disulfidptosis, which may be one of the contributing factors to the differences in patient prognosis.

The five key genes in our risk model are *DLAT*, *STC2*, *POF1B*, *S100A9*, and *CPS1*. Previous studies have found that all of these genes are involved, to varying degrees, in the development and progression of various tumors. *STC2* is a glycoprotein that is expressed in multiple tumor tissues, and several studies have indicated (Cheng et al., 2018; Qie and Sang, 2022) that overexpression of *STC2* promotes cell proliferation and migration, and is associated with tumor growth, invasion, metastasis, and poor prognosis. Inhibiting *STC2* overexpression may be a promising candidate for targeted liver cancer therapy. Lacombe et al. indicated (Lacombe et al., 2006) that *POF1B* is located in a critical region for normal ovarian function, encoding a protein that binds to non-muscle actin filaments and plays an important role in the occurrence and development of premature ovarian failure. Crespi et al. suggest that *POF1B* may function in regulating cell adhesiveness (Crespi et al., 2015). *S100A9* is an important immune-related protein associated with inflammation or cancer, and is believed to promote the occurrence, development, and metastasis of tumors. Zhan et al. reported (Zhan et al., 2023) that HBV-induced activation of *S100A9* triggers the RAGE/TLR4-ROS signaling pathway, leading to the formation of numerous neutrophil extracellular traps, thereby promoting the growth and metastasis of HBV-HCC cells. Inhibiting *S100A9* significantly inhibits the growth and metastatic ability of HCC. *CPS1* encodes the rate-limiting enzyme of the urea cycle, which can more effectively clear ammonia from the body. Aberrant overexpression of the *CPS1* is associated with the rapid proliferation of tumor cells, increasing the activity of pyrimidine biosynthesis through metabolic reprogramming (Zhang et al., 2023). The overexpression of *CPS1* promotes the proliferation of tumor cells by increasing the *de novo* biosynthesis of pyrimidine nucleotides, making *CPS1* a novel target for anti-tumor drugs (Owusu-Ansah et al., 2023). Interestingly, Zhang et al. (2023) found that the urea cycle key enzyme *CPS1* is often low in HCC, and is positively correlated with patient prognosis. The contradictory findings suggest that the impact of *CPS1* on the prognosis of HBV-HCC patients may involve more complex mechanisms, which require further investigation. As one of the core components of PDC, dihydrolipoyl transacetylase (DLAT) is essential for glucose metabolism and the tricarboxylic acid (TCA) cycle (Park et al., 2018). Previous studies have indicated that *DLAT* plays a positive role in the occurrence, development, and metastasis of various tumors (Chen et al., 2022; Li et al., 2023). Increasing evidence suggests that abnormal PDC activity is correlated with the malignant progression of cancer and poor clinical prognosis (Anwar et al., 2021; Jiang et al., 2024). Furthermore, studies have indicated (Tsvetkov et al., 2022) a strong correlation between *DLAT* and cuproptosis. Recently, Li et al. provided the first evidence that (Li et al., 2023) high expression of *DLAT* accelerates the progression of hepatocellular carcinoma, offering a promising therapeutic target for HCC treatment. Additionally, we noted that *DLAT* exhibited the highest coefficient weight in our risk model. Encouraged by these results, we decided to conduct further research on *DLAT*. Analysis of clinical HBV-HCC samples indicates that *DLAT* expression levels are significantly higher in cancerous tissues compared to adjacent normal tissues.

We believe that the differential expression of these five signature genes may play a crucial role in the prognostic differences between genetic subtypes. Therefore, we scored HBV-HCC samples according to the model, obtaining corresponding risk scores. Patients were divided into high-risk and low-risk groups based on the median risk score. Kaplan-Meier survival analysis revealed a significant difference in prognosis between the high-risk and low-risk groups. Additionally, we found significant differences in risk scores between both BHDRGs and genetic subtypes, with higher risk scores observed in subtypes with poorer prognosis. We validated the performance of the signature using both internal and external independent datasets. ROC analysis also demonstrated its strong predictive ability. Compared with previous studies, our BHDRGs signature showed relatively good performance and holds potential as a prognostic tool for predicting outcomes in HBV-HCC patients.

To illustrate the clinical utility of the signature, we constructed a nomogram integrating patient risk scores, age and stage to better predict the survival probability of HBV-HCC patients. By comparing our nomogram with the nomograms developed by Tian, Tang, and Li, and incorporating the results of ROC analysis, calibration curve analysis, and decision curve analysis (DCA), we further validated that our nomogram demonstrates good predictive ability, accuracy, and clinical applicability. Overall, our proposed nomogram is a practical tool for predicting the prognosis of HBV-HCC patients.

The TME is a crucial factor influencing tumor progression and treatment outcomes (Pitt et al., 2016). Therefore, we evaluated the TME of different risk groups. We found that the StromalScore, ImmuneScore, and ESTIMATEScore were significantly higher in the high-risk group compared to the low-risk group. Immunocyte correlation analysis showed that Macrophages M0 and Macrophages M2 were positively correlated with risk scores, whereas T cells CD8 and T cells gamma delta were negatively correlated with risk scores. Previous studies have found (Lu et al., 2023; Tao et al., 2022) that Macrophages M2 promote the development, progression, and metastasis of liver cancer. Macrophages M2 are associated with tumor immune evasion and immunosuppression, often indicating poor patient prognosis (Yang et al., 2020). Raskov et al. indicated (Raskov et al., 2021) that CD8^+^ T cells play a crucial role in immunotherapy, and the relative enrichment of CD8^+^ T cells in the low-risk group facilitates an effective anti-tumor response. T cells gamma delta, a new direction in immunotherapy, have been confirmed by previous research to predict a favorable prognosis when infiltrated in tumors (Gao et al., 2023). However, TIDE score results showed that low-risk patients had significantly higher TIDE scores than high-risk patients, indicating that low-risk patients have higher immune escape potential and are more prone to immune tolerance. Geels SN et al. (Geels et al., 2024) reported that PD-1-mediated activation of CD8^+^ T cells can indirectly promote an increase in Tregs, leading to tumor immune escape. This also explains why, in our study, patients in the low-risk group may face a higher risk of immune escape.

There are ongoing debates regarding whether TMB can accurately predict the efficacy of immunotherapy. McGrail et al. have argued that (McGrail et al., 2021) further research is needed to determine whether a high tumor mutation burden can serve as a universal marker for immunotherapy across all types of solid tumors. Similarly, Anagnostou et al. have noted that (Anagnostou et al., 2022) while TMB holds potential as a broad biomarker for immunotherapy, it cannot yet serve as an independent predictor of immunotherapy response, as patients with low TMB scores may still benefit from treatment. Additionally, methods for detecting TMB require further refinement. Therefore, although the TMB scores between the high- and low-risk groups in our study did not show significant differences, this does not undermine our conclusions. We also concur that further research is necessary to explore both the value and limitations of TMB.

Subsequently, we analyzed the differential expression of immune checkpoint genes between the risk groups. We found that the expression levels of 40 immune checkpoint genes, including the *CD274*, *CD276*, *HAVCR2*, and *CTLA4*, were significantly higher in the high-risk group compared to the low-risk group. The expression results of immune checkpoint genes suggest that the poor prognosis of the high-risk group might be related to HBV-HCC cells evading immune system recognition through activated immune checkpoints. Conversely, high-risk patients may achieve more significant therapeutic effects from immune checkpoint inhibitors. We found that in IMvigor 210, patients who responded significantly to immunotherapy typically had higher risk scores than those with poor immunotherapy outcomes. This trend aligns with our conclusions. In summary, our signature genes could serve as biomarkers for predicting the efficacy of immunotherapy in HBV-HCC patients.

Given the significant role of pharmacotherapy in the treatment of HBV-HCC, we assessed the capacity of signature genes to predict HBV-HCC responses to diverse pharmacological treatments. In the drug sensitivity data provided by the GDSC database, we found differences in drug sensitivity for 75 drugs between the high-risk and low-risk groups. Notably, patients in the high-risk group exhibited higher sensitivity to sorafenib compared to those in the low-risk group. Sorafenib (Huang et al., 2020) is a multi-kinase inhibitor that suppresses tumor cell proliferation in advanced hepatocellular carcinoma and is currently an effective first-line therapy. However, some liver cancer patients develop resistance to sorafenib early in the treatment phase. Witt-Kehati et al. (2018) suggested that HBV, through HBx, participates in the induction of pMAPK14 in liver cancer cells, leading to resistance to sorafenib treatment. We found that low-risk patients had significant sensitivity to palbociclib. Bollard et al. (2017) reported that palbociclib inhibits the proliferation of human liver cancer cell lines by promoting cell cycle arrest. The combination of palbociclib and sorafenib significantly improved the survival rate of HCC samples. Therefore, we believe that the signature genes identified based on BHDRGs have important value for personalized treatment of HBV-HCC patients.

However, our study has some limitations. First, our data were derived from various public databases. Although we applied batch effect corrections, data from different sources inevitably harbor biases that may influence our findings. Thus, further validation using independent cohorts is needed. Additionally, we have only validated the association between DLAT and poor prognosis in HBV-HCC patients. Further research is needed to determine whether other key genes independently or synergistically affect patient outcomes. And we lack more extensive experimental studies to elucidate the specific functions and mechanisms by which these signature genes regulate the biological behavior of HBV-HCC. The efficacy of the immunotherapy predicted by our model was evaluated solely in a cohort of patients with platinum-treated locally advanced or metastatic urothelial carcinoma receiving anti-PD-L1 immunotherapy. Therefore, further validation of our results is needed in a cohort of HBV-related hepatocellular carcinoma patients undergoing immunotherapy. In the future, we will continue to collect more data to assess the accuracy of our model, and we plan to conduct additional *in vivo* and *in vitro* experiments to further explore the regulatory mechanisms of the signature genes in HBV-HCC.

## 5 Conclusion

In summary, our study stratifies HBV-HCC patients into subgroups with different biological behaviors and clinical characteristics using BHDRGs, develops a prognostic model consisting of five signature genes, and constructs a prognostic nomogram. This model can effectively help predict the overall prognosis of patients. Finally, we evaluated the potential of signature genes in chemotherapy and immunotherapy for different risk groups, providing new ideas and directions for future treatment strategies for HBV-HCC patients.

## Data Availability

Publicly available datasets were analyzed in this study. This data can be found here: TCGA-LIHC, https://gdc.cancer.gov/ the GSE45114 dataset and the GSE14520 dataset (GEO, https://www.ncbi.nlm.nih.gov/geo/) the UCSC Xena server (https://xena.ucsc.edu/).
