# Supplementary figures and images for: Exploring prognosis and therapeutic strategies for HBV-HCC patients based on disulfidptosis-related genes
Source: Front Genet. 2025 Jan 15;15:1522484. doi: 10.3389/fgene.2024.1522484 (PMC11774838; doi:10.3389/fgene.2024.1522484)

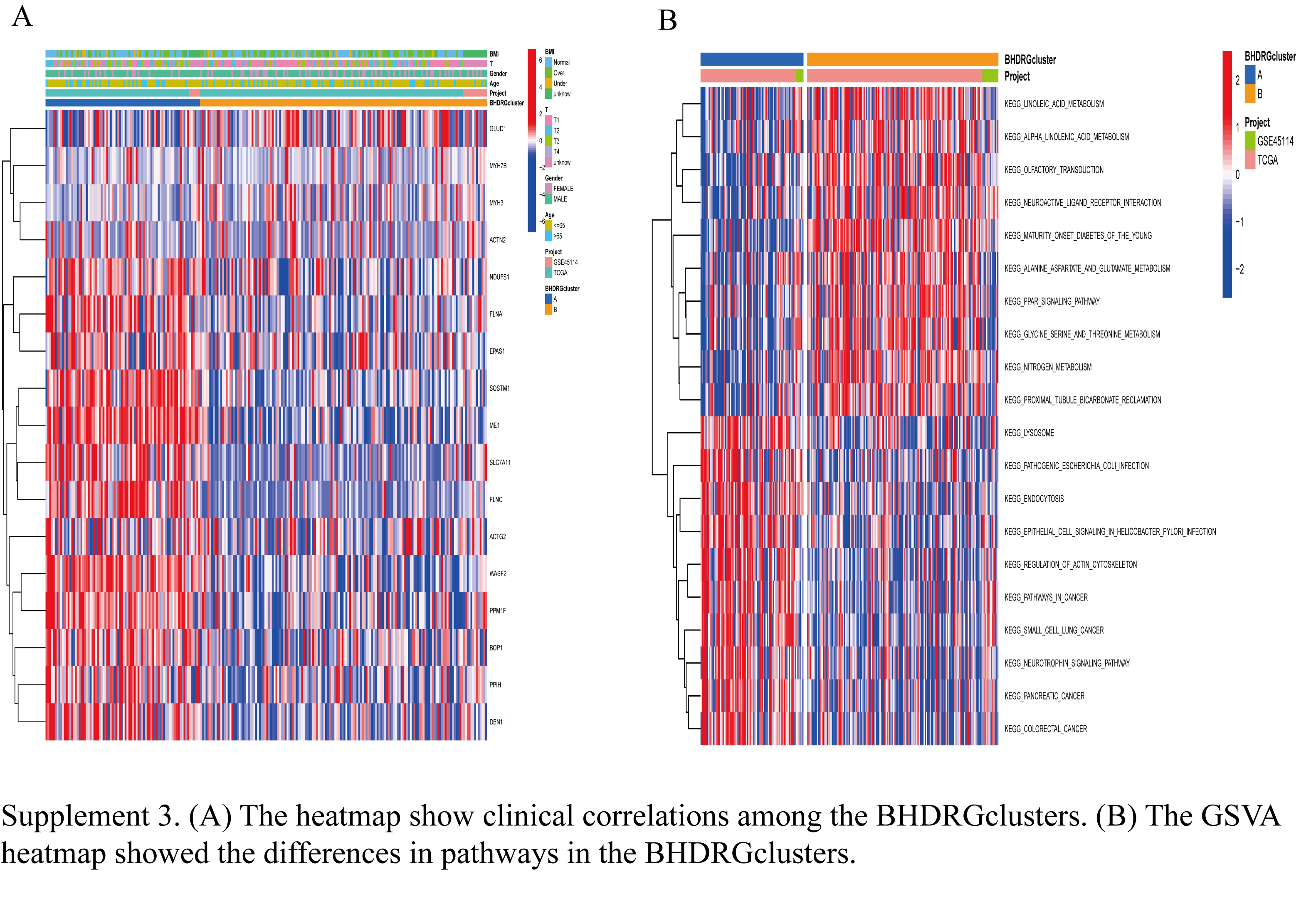

Supplement: Supplementary file 2 [file Image3.tif]

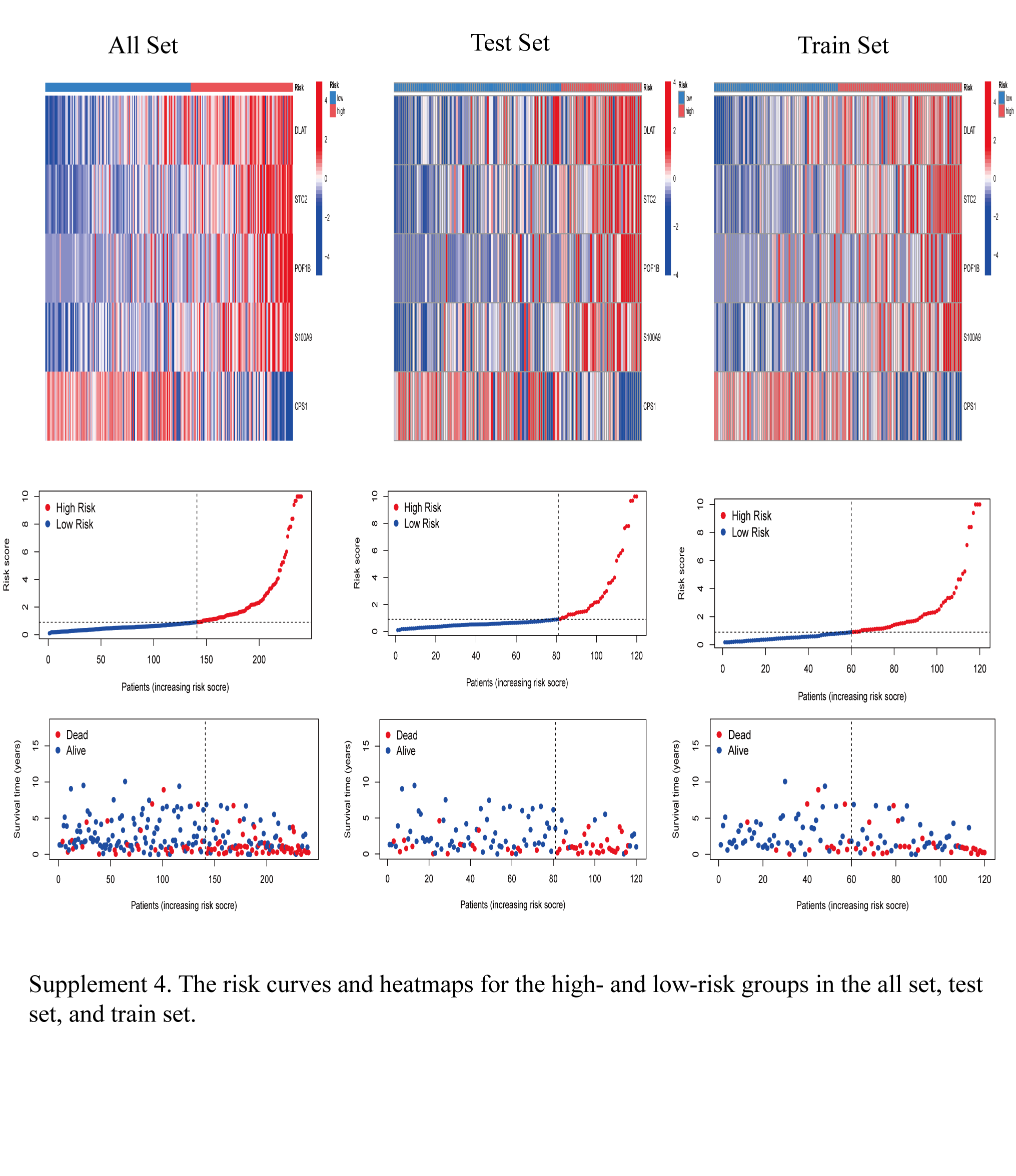

Supplement: Supplementary file 3 [file Image4.tif]

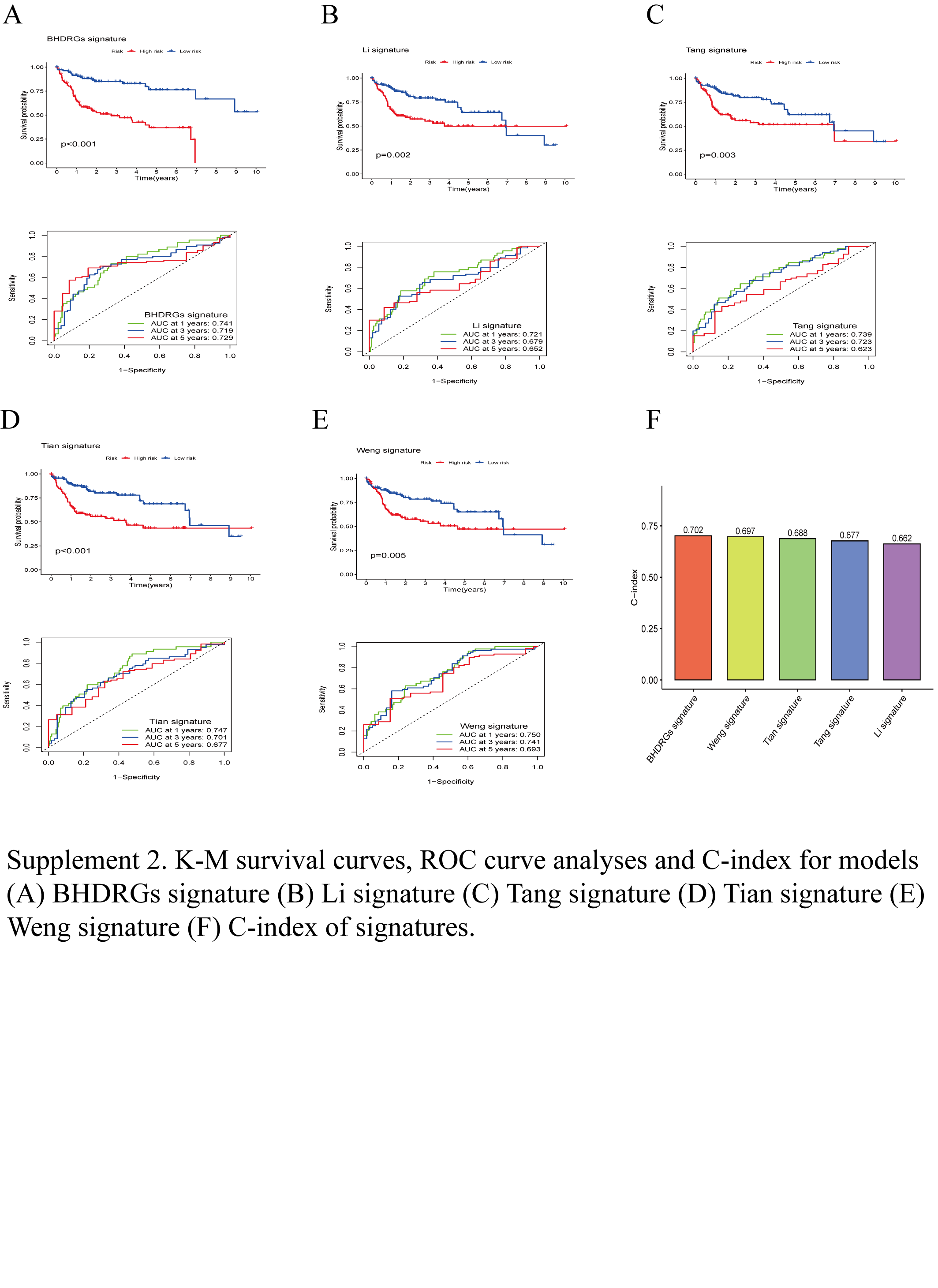

Supplement: Supplementary file 4 [file Image2.tif]

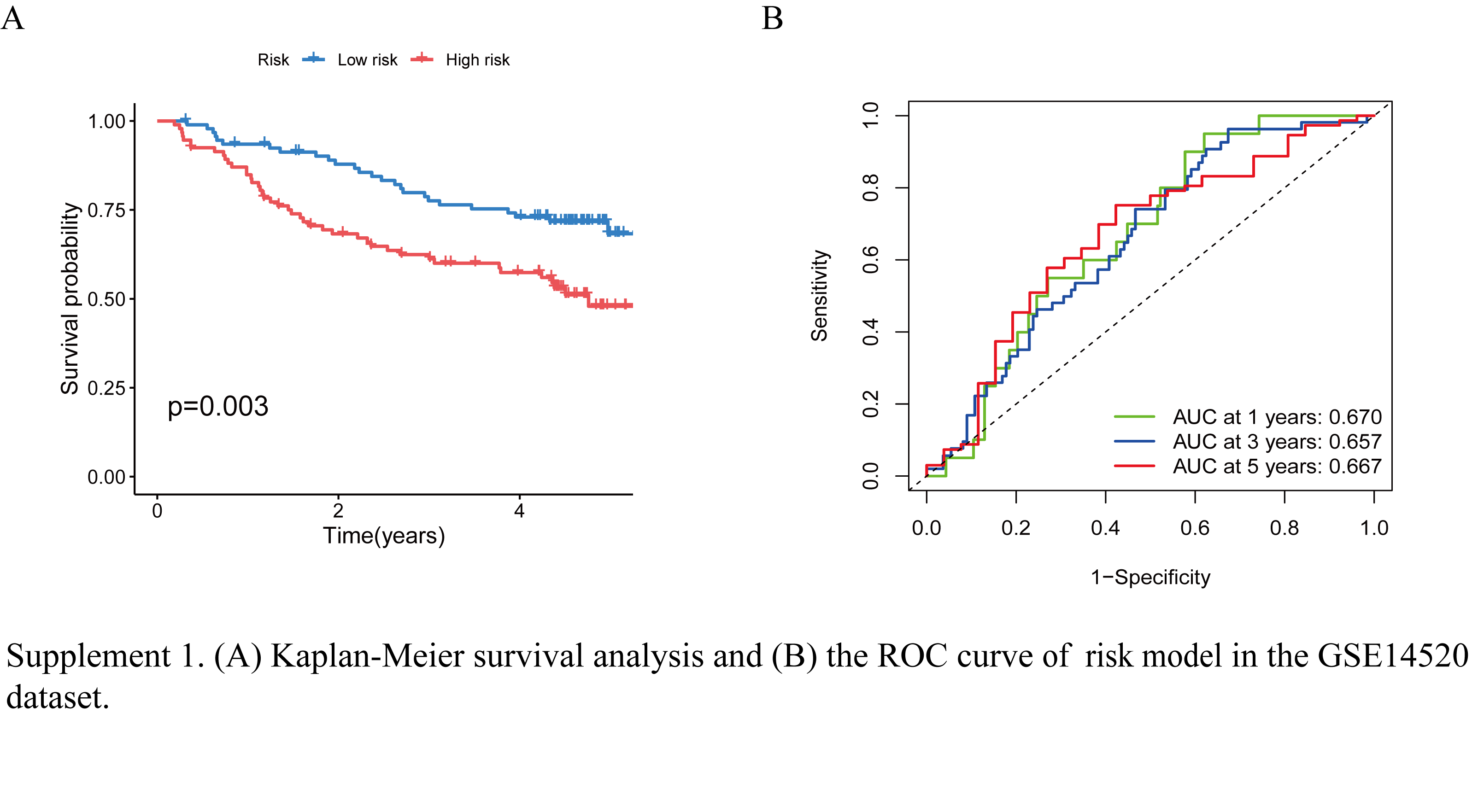

Supplement: Supplementary file 5 [file Image1.tif]

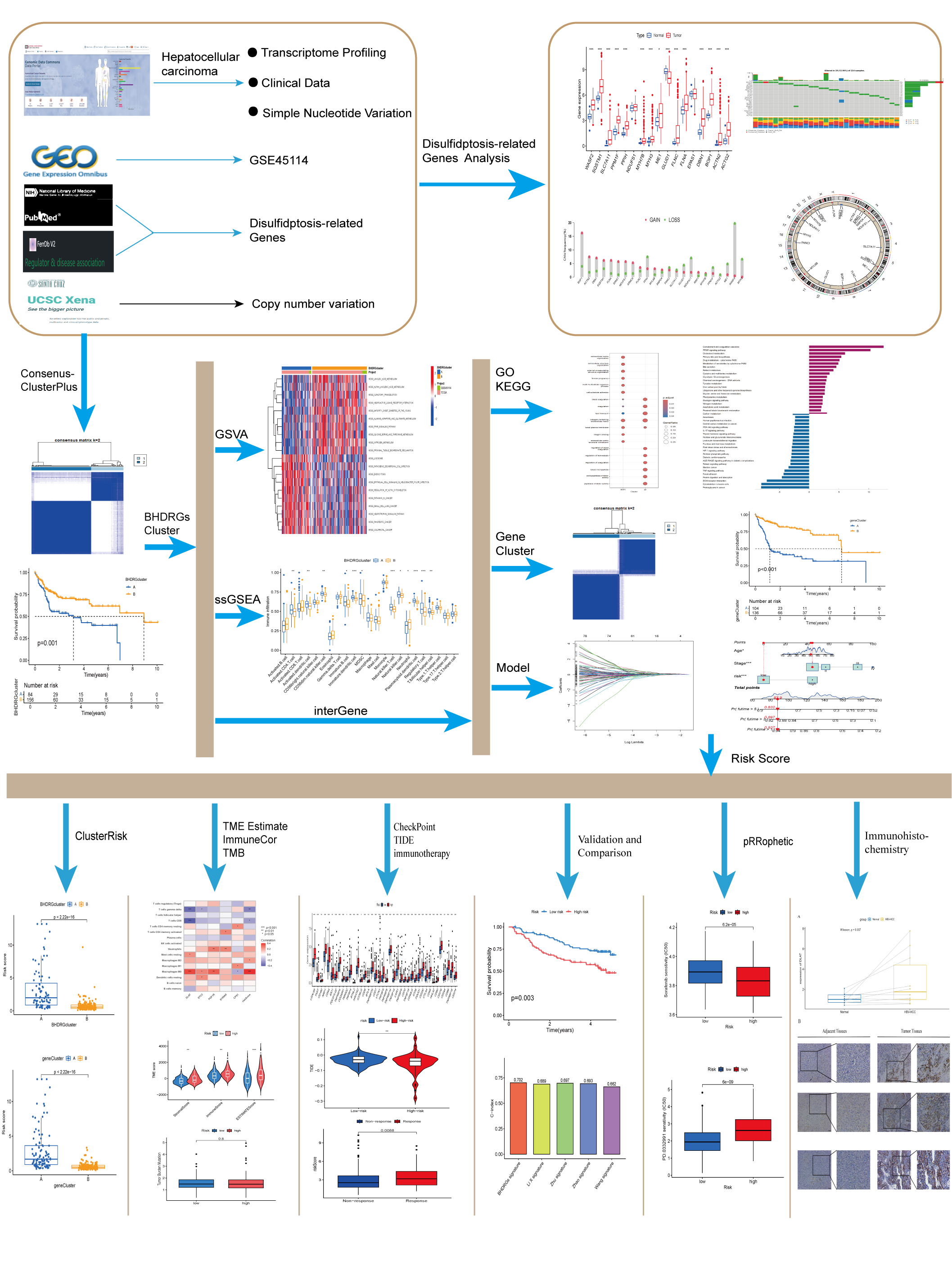

Supplement: Supplementary file 6 [file Supplementaryfile1.tif]

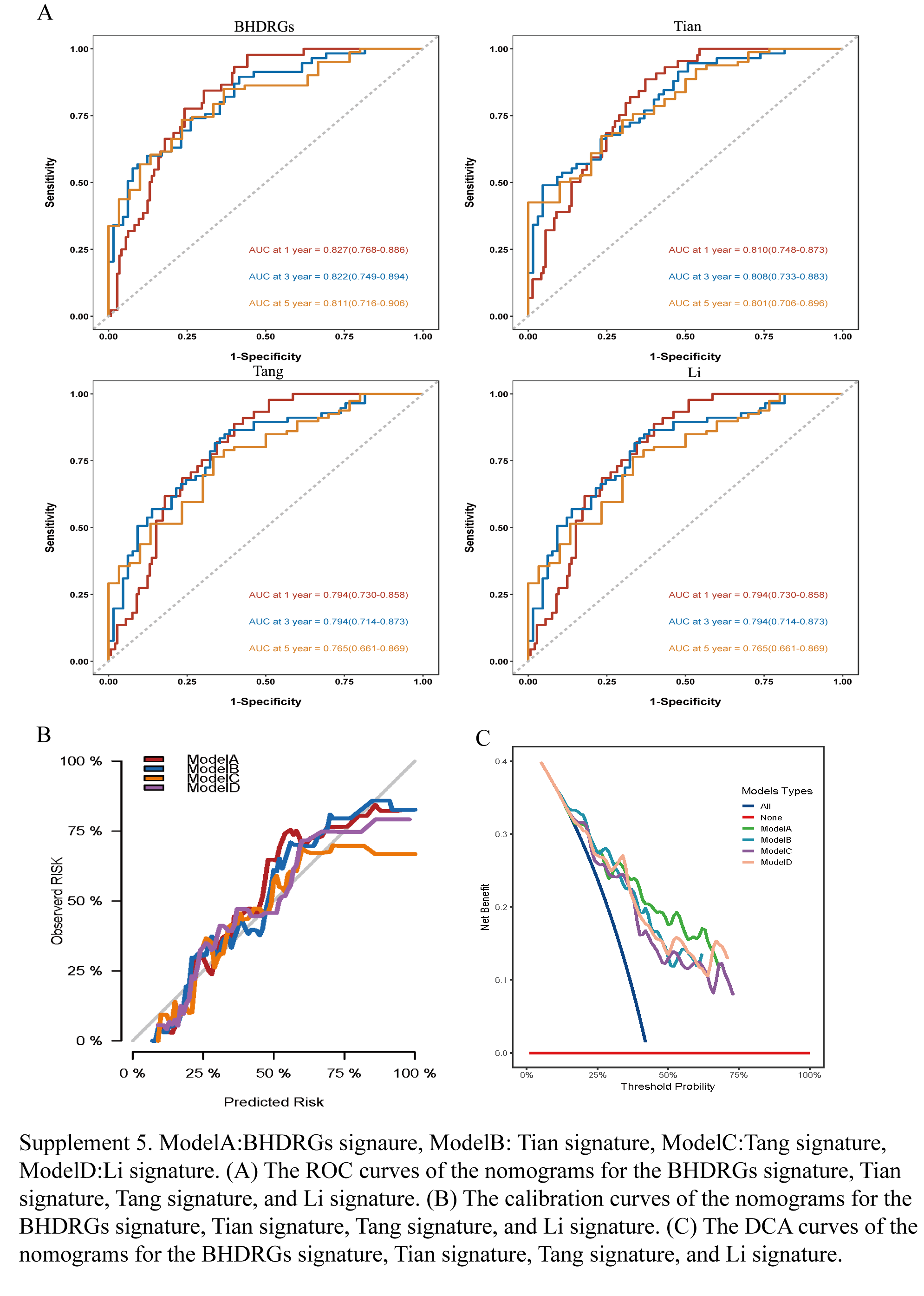

Supplement: Supplementary file 7 [file Image5.tif]
